# Mechanistic Insights into the Effects of Ureas and Monomers on the Ring-Opening Alternating Copolymerization of Epoxides and Anhydrides Catalyzed by Organic Base/Urea

**DOI:** 10.3390/polym16070978

**Published:** 2024-04-03

**Authors:** Lihang Jiang, Yong Wu, Xin Tian, Wanpeng Xue, Hanghang Li, Xiaohui Kang, Bin Li

**Affiliations:** College of Pharmacy, Dalian Medical University, Dalian 116044, China; 15040411200@163.com (L.J.); morph2n@163.com (Y.W.); 18834153277@163.com (X.T.); xuewanpeng22@163.com (W.X.); lhh1762520517@163.com (H.L.)

**Keywords:** aliphatic polyester, organocatalysis, oxygen-containing monomer, theoretical calculation, ring-opening alternating copolymerization

## Abstract

Aliphatic polyester is an important polyester material with good biocompatibility and degradability, which can be synthesized through ring-opening alternating copolymerization (ROAC) of epoxides and anhydrides. Herein, density functional theory (DFT) is used to explore the mechanism of ROAC of epoxides (propylene oxide (PO), styrene oxide (SO), epichlorohydrin (ECH), and cyclohexane oxide (CHO)) and phthalic anhydride (PA) catalyzed by bis(triphenylphosphine) ammonium chloride (PPNCl) and ureas. It was found that the ring-opening polymerization (ROP) of epoxides is the rate-controlling step, and the benzyl alcohol (BnOH) as the initiator has little effect on the polymerization activity, which was consistent with previous experimental results. Calculated comparisons of the ROAC activity of CHO/PA catalyzed by four different ureas indicate that as the Lewis acidity of the urea increased, the energy barriers of the copolymerization increased and the activity decreased. The main reason was that the strong hydrogen-bonding interactions stabilized the key intermediate of the rate-controlling step and inhibited subsequent monomer insertion. Based on this, a series of new ureas with higher catalytic activity were designed by introducing electron-donating substituents. In SO polymerization, increasing the Lewis acidity of urea can improve the SO regioselectivity. In addition, the monomer ECH with CH_2_Cl shows higher activity of ROAC than PO and SO, which could be ascribed to the fact that the strong electron-withdrawing Cl atom stabilizes the transition state in the rate-controlling step and reduces the reaction energy barrier.

## 1. Introduction

Aliphatic polyesters, as an important kind of biocompatible and biodegradable polymer material, can be obtained by biological or chemical synthesis, and have received much attention in the biomedical field because of many advantages such as easy access to materials, modifiability, and structural diversity [1]. The ring-opening alternating copolymerization (ROAC) of epoxides and anhydrides has been proved to be an effective method for the synthesis of structurally diverse polyesters. Anhydrides and epoxides can be obtained from a wide range of sources such as biomass [2]. The ROAC of epoxides/anhydrides was first reported in the 1960s, and a series of catalytic systems were also reported, including inorganic salts, tertiary amines or metal–alkyl catalysts, etc. However, some disadvantages, viz., low selectivity and activity, low molar mass products, and many side reactions, continue to exist in the above catalytic systems [3].

At present, some metal catalysts show high activity, high selectivity, and controllability in the synthesis of polyester [4]. However, metal catalysts tend to produce toxic metal residues, and it is difficult and expensive to remove these impurities from the polymer, severely limiting the application of polyester in biological and medical fields [5]. In contrast, organocatalysts with the advantages of easy synthesis, renewability, low cost, and mild reaction conditions have been successfully applied to the synthesis of polyesters [6]. Among them, the bi-component catalytic systems formed by organic acids and organic bases, mainly including (thio)urea (hydrogen bond donor)/organic base synergistic catalytic systems and Lewis acid–base pairs, have attracted significant attention. It was found that the synergistic effect of organic acids and organic bases can significantly improve catalytic activity and selectivity [7]. Therefore, more and more attempts have been made to develop bi-component organocatalytic systems with high activity and selectivity for the ROAC of epoxides/anhydrides.

In 2018, Li et al. used a bis(triphenylphosphine) ammonium chloride (PPNCl)/triethyl borane (TEB) organic bimolecular catalytic system to catalyze the ROAC of epoxides/anhydrides, and synthesized polyesters with perfect alternating structures [8]. That study demonstrates the great potential of PPNCl to efficiently catalyze the ROAC of epoxides and anhydrides, and also indicates that the addition of organic acid TEB can significantly improve catalytic activity and selectivity compared with the single component catalyst. Meng et al. reported that PPNCl/urea catalyzed the ROAC of different epoxides and anhydrides, and found that the TOF value is six times higher than that of pure PPNCl catalysts, which is even comparable to metal-based catalysts. The catalytic activity increases gradually as the acidity of urea decreases [9]. Later, they used a PPNCl/urea system to catalyze the copolymerization of epoxides/anhydride/lactone to synthesize triblock copolyesters. When benzenedimethanol (BDM) was involved in the reaction as the initiator, the alternating copolymerization of epoxides/anhydride was carried out first, and the homopolymerization of epoxides did not occur even in the presence of excessive epoxides [10]. The results showed that the addition of initiator BDM did not increase the activity of ROAC of epoxides/anhydride, but changed the initial structure of the chain initiation stage. In the absence of an initiator, the polymerization begins with the ring-opening of epoxides, while in the presence of an initiator, the reaction begins with the ring-opening of anhydrides. Although the PPNCl/urea system has been successfully applied to the ROAC of epoxides and anhydrides and shows excellent catalytic activity, the mechanism details of polymerization and the origin of the influence of urea acidity on polymerization activity need to be further uncovered.

Density functional theory (DFT) studies have been applied to the ROAC of epoxides and anhydrides catalyzed by organic catalysts. Most calculations have focused on studying the mechanisms of TEB combined with organic base systems. For instance, Li et al. performed DFT studies on the ring-opening copolymerization (ROCOP) mechanism of epoxide/anhydride/CO_2_ with a bi-component catalyst PPNCl/TEB, and clarified the origin of selectivity of copolymerization [11]. In 2023, they computationally studied the mechanism of the copolymerization of CO_2_/epoxide/acid anhydride to uncover the reason behind the chemical selectivity of monomers, and to explain the influence of the molar ratio of phosphazene (C_3_N_3_-Py-P_3_)/TEB on chemical selectivity. In addition, they also confirmed that the strong proton acceptance of C_3_N_3_-Py-P_3_ is important for initiating the reaction, because it polarizes the BDM to generate an alkoxide as a nucleophile [12]. Therefore, DFT studies can be used to clarify the mechanisms of copolymerization and to explore chemical issues, such as reaction activity and selectivity, in depth.

In the present work, we carried out DFT computations on the mechanism of the ROAC of epoxides and anhydrides by bi-component organocatalyst PPNCl/urea, in order to explore the influences of the initiator and the Lewis acidity of the urea on catalytic activity and regioselectivity, and to make further theoretical modifications for urea structures. In addition, the origin of side-chain substituents of epoxide affecting the polymerization activity was also investigated. This is the first theoretical calculation study on the ROAC of epoxides and anhydrides catalyzed by PPNCl/urea. Various factors affecting the reactivity and selectivity were analyzed in depth, and the catalytic activity of the catalytic system was theoretically improved; as such, the calculation provides some valuable theoretical insights for experiment.

## 2. Computational Details

All calculations were carried out with density functional theory (DFT) using the Gaussian 16 [13] software package. Geometry optimizations, as well as frequency calculations of intermediates (INT) and transition states (TS), were performed with the B3LYP [14] functional and 6-31G(d,p) basis sets. Each optimized structure was subsequently analyzed by harmonic vibration frequencies for the characterization of a minimum (Nimag = 0) or a transition state (Nimag = 1) and to provide thermodynamic data. To further obtain Gibbs free energies (kcal/mol) with a high degree of accuracy, single-point calculations were then done under the B3LYP-D3BJ [15]/6-311+G(2d,p) level in toluene solvent with the SMD [16] solvation model. The 3D molecular structures displayed in this paper were drawn by using CYLview v1.0 [17].

## 3. Results and Discussion

### 3.1. The Mechanism of the ROAC of CHO/PA Catalyzed by PPNCl/Urea

**Figure 1:**
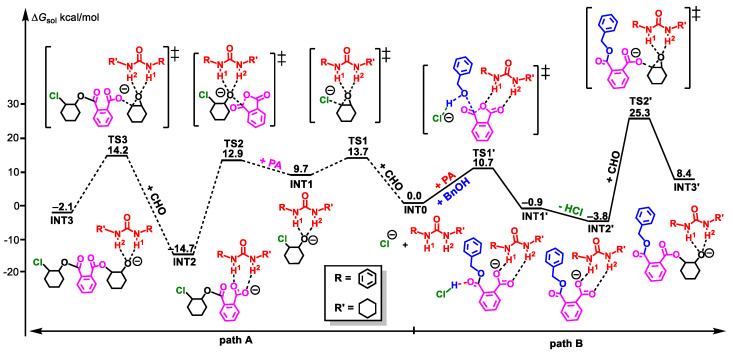
Energy profiles of ROAC of CHO/PA catalyzed by PPNCl/U2 (path A: no BnOH as initiator; path B: BnOH as initiator “‡” represents transition state).

The mechanism of the ROAC of cyclohexane oxide (CHO)/phthalic anhydride (PA) catalyzed by PPNCl/U2 was calculated. In order to simplify the calculation process, PPNCl was replaced by Cl^−^ with nucleophilic activity, and the reaction mechanisms (Figure 1) of both no BnOH as the initiator (path A) and BnOH as the initiator (path B) were considered.

In path A, the Cl^−^ as a nucleophile attacks the carbon (C) atom of the epoxy group in epoxide CHO activated by urea U2 through double hydrogen(H)-bonding interactions to lead to the ring-opening reaction of CHO. This step needs to overcome a Gibbs free energy barrier of 13.7 kcal/mol (**TS1**) to generate an intermediate **INT1**. Then, the alkoxy anion attacks PA via a transition state **TS2** with an energy barrier of 3.2 kcal/mol to produce an intermediate **INT2** containing a CHO/PA copolymer unit. The H1 and H2 atoms of molecule U2 in **INT2** stabilize the carboxylate anion formed by PA through H-bonding interactions. Then, the nucleophilic attack of the carboxylic anion on the second CHO takes place via a **TS3** with an energy barrier of 28.9 (14.2 − (−14.7)) kcal/mol, generating the alternating copolymerization product **INT3**, which is the rate-controlling step of the whole reaction process.

In path B, Cl^−^ grabs the H of BnOH to generate an alkoxy anion with nucleophilic activity, then this alkoxy anion attacks the carbonyl C atom of PA activated by U2 to realize the ring-opening of PA. This process goes through a **TS1’** with an energy barrier of 10.7 kcal/mol. After that, the intermediate **INT1’** with a carboxylate anionic terminal stabilized by H1 and H2 atoms of urea was obtained. Then, one molecule of HCl was removed to form the **INT2’** by an energy release of 2.9 kcal/mol. Finally, the nucleophilic attack of the carboxylic anion on CHO overcomes an energy barrier of 29.1 (25.3 − (−3.8), **TS2’**) kcal/mol to give the copolymer product **INT3’**.

In contrast, in the absence of BnOH, the polymerization reaction catalyzed by the PPNCl/urea system starts from the ring-opening of CHO, and while BnOH was used as the initiator, the ring-opening of PA occurred first, as reported in previous experiments [9,10]. The rate-controlling step in both situations is the ring-opening process of CHO, and they both show similar energy barriers (28.9 vs. 29.1 kcal/mol), suggesting that these two cases have similar copolymerization activities. This is consistent with previous experimental results.

### 3.2. Catalyst Design

**Figure 2:**
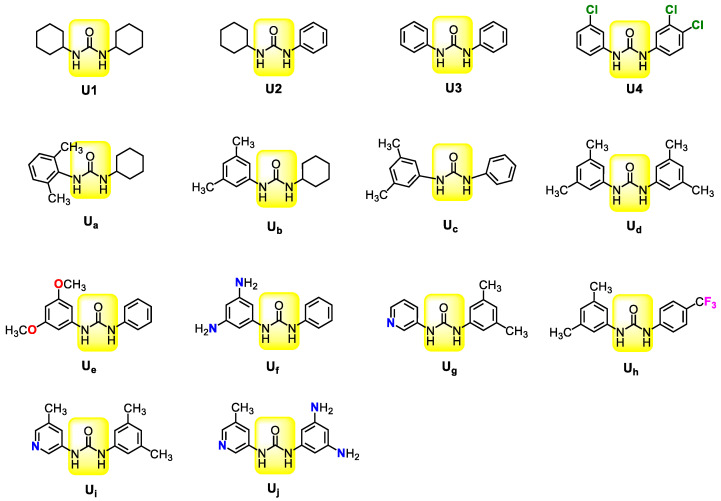
Ureas with different structures.

Meng et al. reported the ROAC of CHO/PA catalyzed by four different ureas with different Lewis acidity in coordination with PPNCl (Figure 2). The results showed that in the catalysis of dicyclohexyl substituted U1 (p*K*a = 26.9), the conversion rate of PA reached 76% in 10 min, which was the highest catalytic activity level. When one cyclohexyl group in U1 was substituted by a phenyl group (U2, p*K*a = 22.8/25.1), the conversion rate of PA was 62%. And the conversion rate of diphenyl-substituted U3 (p*K*a = 20.8) was 38% after 20 min. Based on U3, when three Cl atoms were introduced into the *ortho*- and *meso*-sites of phenyls (U4, p*K*a = 19.2/19.0), the conversion rate (28%, 20 min) decreased. Therefore, the experimental results showed that the catalytic activity decreased with the increase of urea acidity. To clarify how the structures of ureas regulate polymerization activity, the ROACs of CHO and PA—catalyzed by U1, U2, U3, and U4—with PPNCl were calculated.

**Figure 3:**
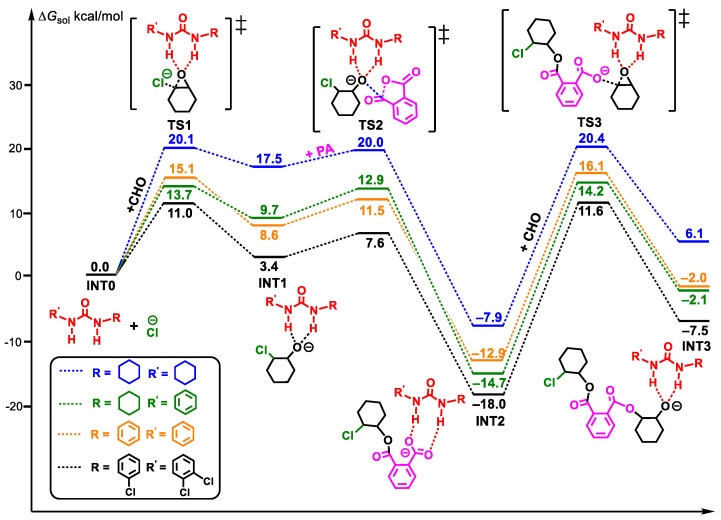
Energy profiles of the ROAC of CHO/PA, catalyzed by U1 (blue), U2 (green), U3 (orange), and U4 (black), with PPNCl (“‡” represents transition state).

As shown in Figure 3, all polymerization processes follow the same mechanism as that of path A in Figure 1, and the energy barriers of CHO insertion as the rate-controlling step for U1, U2, U3, and U4 are 28.3, 28.9, 29.0, and 29.6 kcal/mol, respectively. Therefore, the above results show that with the increase of urea acidity, the rate-controlling step barrier increases and the catalytic activity decreases, showing a good agreement with the previously mentioned experimental phenomena.

**Figure 4:**
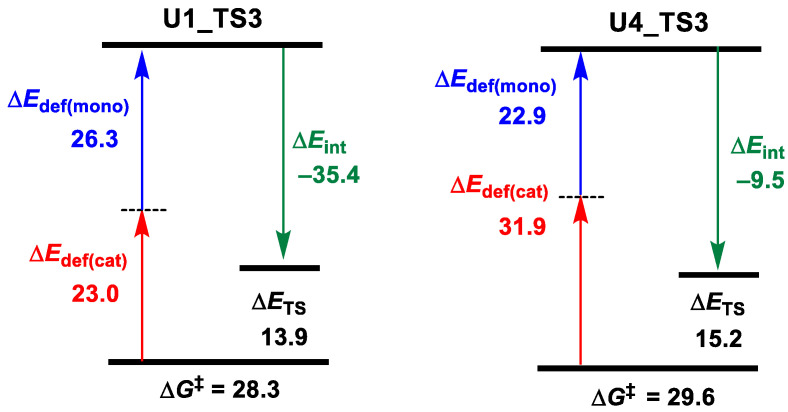
Energy (in kcal/mol) decomposition analyses for U1_TS3 and U4_TS3 (“‡” represents energy barrier).

In order to further clarify the relationship between polymerization activity and urea acidity, energy decomposition analyses for **TS3** in the U1 and U4 cases were performed. Among them, the fragments mono and cat represented the monomer CHO and the remaining catalyst parts in **TS3**, respectively. As shown in Figure 4, the total deformation energy Δ*E*_def_ of **U1_TS3** is 49.3 (26.3 + 23.0) kcal/mol, and the interaction energy Δ*E*_int_ between CHO and catalyst is −35.4 kcal/mol, so the Δ*E*_TS_ = Δ*E*_def_ + Δ*E*_int_ = 13.9 kcal/mol. In contrast, the bigger deformation energy Δ*E*_def_ (22.9 + 31.9 = 54.8 kcal/mol) of **U4_TS3** completely shields the advantage of the stronger interaction energy (Δ*E*_int_ = −39.5 kcal/mol), resulting in higher Δ*E*_TS_ (15.2 vs. 13.9 kcal/mol). Therefore, the lower activity of U4 than U1 may be due to the larger steric repulsion between CHO and the catalyst.

**Figure 5:**
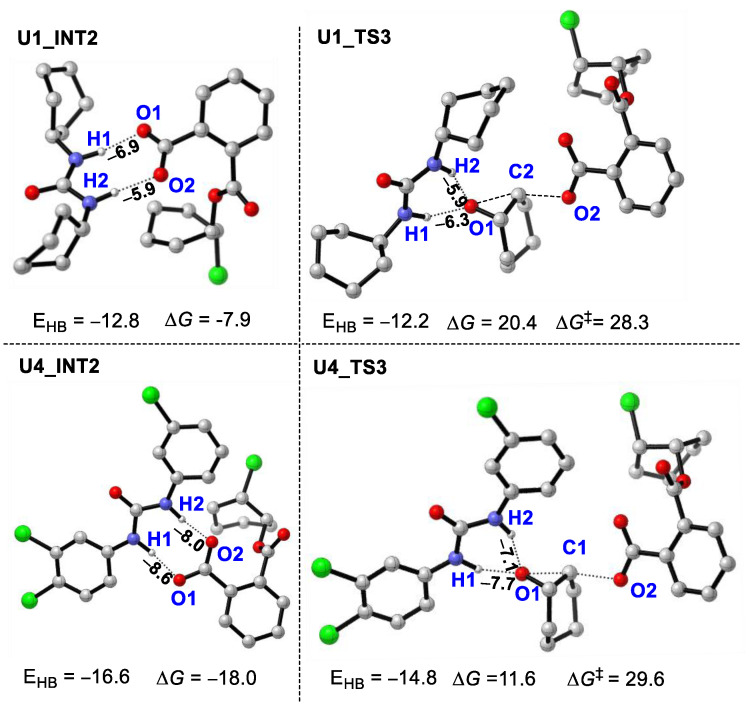
The optimized structures of INT2 and TS3 under U1 and U4, respectively (the energy of hydrogen bonds is shown in black words, E_HB_ represents the total energy of hydrogen bonds, energy is in kcal/mol, “‡” represents energy barrier).

In order to further clarify the origin of the higher activity of U1 than U4, the geometric structures of **INT2** and **TS3** were analyzed. As shown in Figure 5, the total energy of the hydrogen bonds (E_HB_) H1—O1 and H2—O2 in **U4_INT2** is −16.6 kcal/mol, which is greater than that of **U1_INT2** (E_HB_ = −12.8 kcal/mol). Likewise, the E_HB_ of the hydrogen bonds H1—O1 and H2—O1 in **U4_TS3** (E_HB_ = −14.8 kcal/mol) is greater than that of **U1_TS3** (E_HB_ = −12.2 kcal/mol). This shows that the H-bonding interactions between H1/H2 in the stronger acidity U4 and the O at the chain end is stronger than that in U1’s case, and the acidity of the urea has a greater influence on H-bonding interactions in **INT2** (∆E_HB_ = −16.6 − (−12.8) = −3.8 kcal/mol) than that of **TS3** (∆E_HB_ = −14.8 − (−12.2) = −2.6 kcal/mol). Due to the stronger acidity of U4, the H-bonds in **U4_INT2** are enhanced, making **U4_INT2** too stable. Therefore, the CHO insertion needs to break stronger H-bonding interactions to overcome the greater deformation energy, so the insertion energy barrier increases.

According to above results, it was concluded that reducing the acidity of urea can improve its catalytic activity. Based on U2 and U3, therefore, the electron-donating substituents were used to design ten structures of Ua~Uj (Figure 2). The energies of their corresponding intermediates **INT2** (∆G_INT2_), transition states **TS3** (∆G_TS3_), and energy barriers (∆G^‡^) were calculated (Table 1).

Firstly, two methyls (CH_3_) were introduced to the *ortho*- and the *meso*-sites of the phenyl group in U2 to give Ua and Ub, respectively. Under these two catalysts, the energy barriers of rate-controlling steps were 28.5 and 27.3 kcal/mol, respectively. In comparison, the energy barrier in the catalysis of Ub was lower than that in U1 (28.3 kcal/mol), suggesting that higher catalytic activity can be obtained by introducing electron-donating substituents to the *meso*-sites of phenyl groups. Based on U3, different numbers of CH_3_ or trifluoromethyl (CF_3_) groups were introduced to the *meso*-site of the phenyl to lead to new ureas Uc, Ud, and Uh. Their corresponding energy barriers are 26.9, 27.0, and 27.8 kcal/mol, respectively. Although Ud has more electron-donating CH_3_ groups, its corresponding energy barrier is higher than that of Uc. This energy barrier (26.9 kcal/mol) of Uc with two unsaturated phenyls is even lower than that of Ub (27.3 kcal/mol) with one cyclohexyl. Therefore, the aforementioned results confirm that it is more beneficial to improve catalytic activity through introducing both two phenyls and an electron-donating substituent at the *meso*-site of only one of the phenyl groups.

Then, two methyls in Uc are replaced by methoxy(OMe) or amino (NH_2_) groups to obtain Ue and Uf with energy barriers of 28.0 and 24.5 kcal/mol. Obviously, the strong electron-donating ability of NH_2_ significantly reduces the reaction energy barrier and improves the polymerization activity. In addition, replacing a phenyl group of Uc with pyridyl was also attempted in order to obtain Ug, during which the rate-controlling step barrier was 27.6 kcal/mol. This suggests that the substitution of a phenyl group with pyridyl reduces the catalytic activity. However, introducing one CH_3_ (Ui) to the *meso*-site of pyridyl can increase the catalytic activity (26.6 vs. 27.6 kcal/mol). Finally, the Uj obtained by replacing the two CH_3_ groups of phenyl of Ui with NH_2_ groups indicates an energy barrier of 24.8 kcal/mol, which is close to that of Uf (24.5 kcal/mol). Overall, the use of NH_2_ as a strong electron donor can significantly improve the catalytic activity of ureas.

### 3.3. The ROAC of Different Epoxides and PA Catalyzed by PPNCl/Urea

In order to explore the influence of different epoxides on catalytic activity, we considered the ROAC of propylene oxide (PO), styrene oxide (SO), and epichlorohydrin (ECH) with PA by PPNCl/urea (U1).

#### 3.3.1. Regioselective ROAC of SO and PA

**Figure 6:**
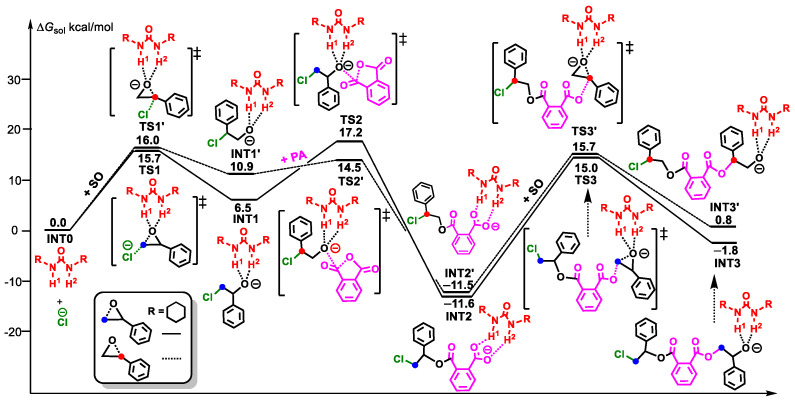
Energy profiles of ROAC of SO and PA at methylene (solid line) and methine (dotted line) sites catalyzed by PPNCl/U1 (“‡” represents transition state).

Due to the electronic properties of SO, nucleophilic attacks can occur at two sites, viz., the methylene site and the methine site, due to different regioselectivity during the insertion process. To further compare the copolymerization activity of different epoxides, here we firstly discuss the regioselectivity of SO insertion in the ROAC of SO and PA by PPNCl/U1 (Figure 6).

Firstly, the Cl^−^ of PPNCl attacks the methylene and methine sites of SO; meanwhile, the hydrogen atoms H1 and H2 of the urea stabilize the alkoxy anion formed by the ring-opening of SO through H-bonding interactions. These two pathways take place through **TS1** and **TS1′**, with energy barriers of 15.7 and 16.0 kcal/mol, to generate **INT1** and **INT1′**, respectively. The almost-identical energy barriers indicate that the activity levels of the ROP of SO at the two sites are similar in kinetics. Then, the alkoxy anions generated by the ring-opening of SO attack the carbonyl C atom of PA and go through **TS2** and **TS2′**, with the energy barriers of 10.7 (17.2 − 6.5) kcal/mol and 3.6 (14.5 − 10.9) kcal/mol, respectively, leading to the ring-opening of PA to form intermediates **INT2** and **INT2′**, with similar energies (11.6 vs. 11.5 kcal/mol). Finally, the carboxylic anion attacks the methylene and methine sites of SO via **TS3** and **TS3’**, with energy barriers of 26.6 (15.0 − (−11.6)) and 27.2 (15.7 − (−11.5)) kcal/mol, to form **INT3** and **INT3’**, respectively. The energy barrier gap of these two pathways is only 0.6 kcal/mol. Therefore, it is speculated that the PPNCl/U1 catalytic system shows poor regioselectivity for the ROAC of SO and PA.

In order to further improve the regioselectivity of SO insertion, U1, U2, U3, and U4 were selected to catalyze the ROAC of SO and PA. The rate-controlling step **INT2** (**INT2’**) → **TS3** (**TS3’**) was calculated. Δ*G*^‡^_(TS/TS’)_ represents the energy barriers of rate-controlling steps, and the selectivity is decided by the energy barrier gap (ΔΔ*G*^‡^ = Δ*G*^‡^_(TS’)_ − Δ*G*^‡^_(TS)_) of two pathways. The computational results are summarized in Table 2.

The results show that the energy barriers (Δ*G*^‡^_(TS)_ and Δ*G*^‡^_(TS’)_) of the two pathways of U2 are 27.9 and 28.6 kcal/mol, respectively, and the ΔΔ*G*^‡^ is 0.7 kcal/mol. The Δ*G*^‡^_(TS)_ and Δ*G*^‡^_(TS’)_ in U3’s situation are 28.3 and 29.5 kcal/mol, respectively, and the ΔΔ*G*^‡^ is 1.2 kcal/mol. The Δ*G*^‡^_(TS)_ and Δ*G*^‡^_(TS’)_ in U4’s case are 29.4 kcal/mol and 31.0 kcal/mol, respectively, and the ΔΔ*G*^‡^ is 1.6 kcal/mol. These results indicate that with the increase of urea acidity, both the energy barriers of the two ring-opening manners and the ΔΔ*G*^‡^ increase, suggesting that increasing the acidity of ureas can improve the regioselectivity of SO insertion in the ROAC of SO/PA.

#### 3.3.2. The ROAC of PO, SO, and ECH with PA Catalyzed by PPNCl/U1

**Figure 7:**
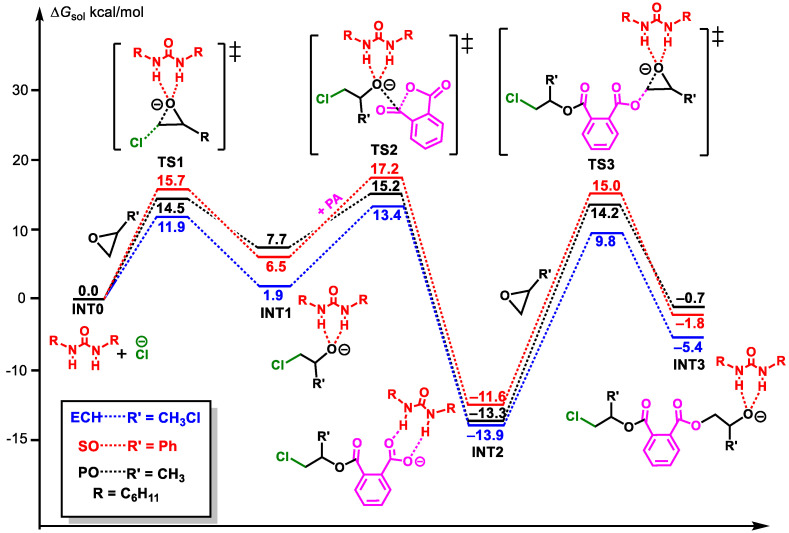
Energy profiles of the ROAC of PO, SO, and ECH with PA catalyzed by PPNCl/U1 (“‡” represents transition state).

Herein, the copolymerizations of three epoxides—PO, SO, and ECH with PA, catalyzed by PPNCl/U1—were calculated in order to compare their activity. As shown in Figure 7, the polymerization processes of the three epoxides follow the same mechanism as CHO, and the processes of the second epoxide insertion are the rate-controlling steps. The energy barriers of the rate-controlling steps of PO, SO, and ECH were 27.5 (14.2 − (−13.3)), 26.6 (15.0 − (−11.6)), and 23.7 (9.8 − (−13.9)) kcal/mol, respectively. The energy barriers of PO, SO, and ECH were lower than that of CHO (28.3 kcal/mol). Therefore, PPNCl/U1 showed higher copolymerization activity for the epoxides PO, SO, and ECH. By contrast, the energy barrier of ECH is the lowest, followed by SO. Therefore, the introduction of substituents such as phenyl or chlorine can improve the polymerization activity in comparison with PO.

**Figure 8:**
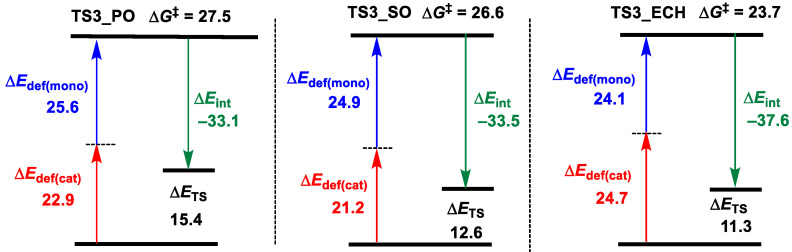
Energy (kcal/mol) decomposition analyses for TS3_PO, TS3_SO, and TS3_ECH (“‡” represents energy barrier).

In order to further explore the reasons for the differences in polymerization activity between the three monomers, the rate-controlling step transition states, **TS3**, of three monomers—PO, SO, and ECH—were analyzed by energy decomposition (Figure 8). Among them, the fragments mono and cat represented the monomer and the remaining catalyst parts in **TS3**, respectively. The results show that the interaction energies (∆*E*_int_) between monomer and catalyst fragments in **TS3_PO**, **TS3_SO**, and **TS3_ECH** are −33.1, −33.5, and −37.6 kcal/mol, respectively, and the total deformation energies (∆*E*_def_) of the two fragments are 48.5 (22.9 + 25.6), 46.1 (21.2 + 24.9), and 48.8 (24.7 + 24.1) kcal/mol, respectively. Therefore, the energies ∆*E*_TS_ corresponding to the three monomers are 15.4 (−33.1 + 48.5), 12.6 (−33.5 + 46.1), and 11.3 (−37.6 + 48.8) kcal/mol, respectively, showing a good agreement with the energy barriers. By contrast, the stability of **TS3_SO** in comparison with **TS3_PO** was mainly ascribed to the lower deformation (46.1 vs. 48.5 kcal/mol). Meanwhile, the stronger interaction (ECH vs. PO: 37.6 vs. 31.1 kcal/mol) between ECH and the catalyst fragments stabilizes **TS3_ECH**, leading to the higher copolymerization activity of ECH and PA in comparison with PO.

**Figure 9:**
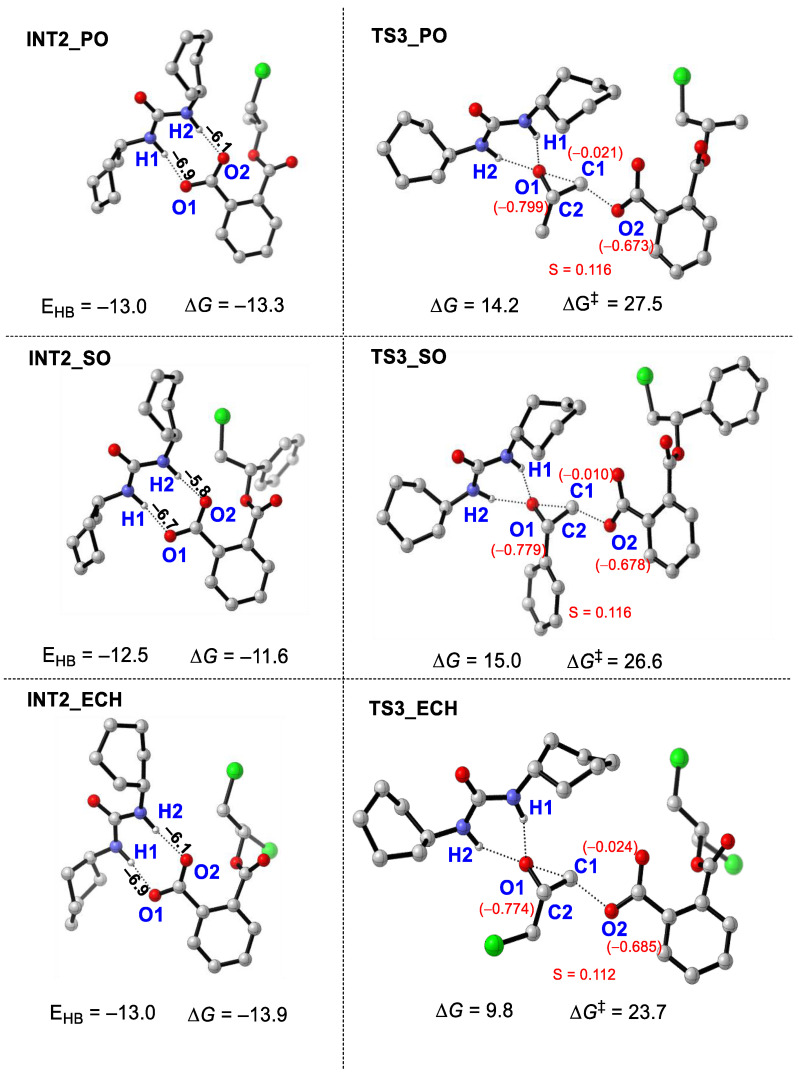
The optimized geometrics of the INT2 and TS3 of PO, SO, and ECH (the NBO charge is shown in red words; the energy of hydrogen bonds is shown in black words; E_HB_ represents the total energy of hydrogen bonds; S represents the variance of absolute value of the NBO charge; energy is in kcal/mol “‡” represents energy barrier).

Further geometric analyses for intermediate **INT2** and transition state **TS3** in PO, SO, and ECH cases were carried out. As shown in Figure 9, the total energy of hydrogen bonds (E_HB_ = −13.0 kcal/mol) H1—O1 and H2—O2 in **INT2_PO** is bigger than that in **INT2_SO** (E_HB_ = −12.5 kcal/mol), suggesting that stronger H-bonding interactions stabilize **INT2_PO**. As we all know, the dispersion of charge will affect the stability of the structure. Therefore, the NBO charges of the atoms at the reaction centers (O1, O2, C1) of **TS3_PO** and **TS3_SO** were investigated, and the variance S of the absolute value of the NBO charge was calculated. Generally, the smaller the value of S, the more uniform the charge distribution and the lower the energy of the structure [18]. The S value (about 0.116) of **TS3_SO** is the almost same as that of **TS3_PO**, indicating that the stability of the two TSs is similar. Further PO insertions need to break the stronger H-bonding interactions in **INT2_PO**, so the increased stability of **INT2_PO** inhibits subsequent monomer insertion, leading to lower copolymerization activity.

Then, the total energy of the hydrogen bonds (E_HB_ = −13.0 kcal/mol) H1—O1 and H2—O2 in **INT2_ECH** is the same as that of **INT2_PO**, suggesting that the stability of the two INTs is similar. However, the S value in **TS3_ECH** (about 0.112) is smaller than that in **TS3_PO** (about = 0.116), suggesting that the strong electron-absorbing Cl atoms enhance the structural stability of the rate-controlling transition state by improving the uniformity of the charge distribution in **TS3**, thus reducing the reaction energy barrier and increasing the activity of copolymerization.

## 4. Conclusions

The ROACs of epoxides and anhydrides (PA) catalyzed by an organic two-component PPNCl/urea system were studied through theoretical calculation. The results showed that the rate-controlling step of copolymerization occurs during epoxide insertion, and benzyl alcohol as an initiator has little effect on catalytic activity. Urea as the H-bonding donor plays the role of the activating monomer and stabilizes the chain end of the copolymer. Strongly acidic ureas make the key intermediates in rate-controlling steps more stable, inhibiting the epoxide insertion, so the ROAC activity is reduced. Based on this, several kinds of ureas with higher catalytic activity levels have been designed by introducing electron-donating groups, viz., methyl or amino, to the *meso*-sites of the phenyls of ureas. Meanwhile, increasing the acidity of ureas can improve the regioselectivity of SO insertion. Compared with PO and SO, ECH has highest ROAC activity, because the strong electron-absorbing Cl atom of ECH stabilizes the rate-controlling transition state to further reduce the reaction energy barrier. The above findings are expected to provide some theoretical information for designing and developing highly efficient organocatalysts for the synthesis of new polyester materials through the ROACs of epoxides and anhydrides.

## Figures and Tables

**Table 1:** Energies (kcal/mol) of intermediates and transition states in different ureas.

Un	∆G_INT2_	∆G_TS3_	∆G^‡^
U1	−7.9	20.4	28.3
U2	−14.7	14.2	28.9
U3	−12.9	16.1	29.0
U4	−18.0	11.6	29.6
Ua	−13.2	15.3	28.5
Ub	−12.6	14.7	27.3
Uc	−10.9	16.0	26.9
Ud	−9.8	17.2	27.0
Ue	−13.5	14.5	28.0
Uf	−11.2	13.3	24.5
Ug	−12.4	15.2	27.6
Uh	−13.1	14.6	27.8
Ui	−11.3	15.3	26.6
Uj	−11.8	13.0	24.8

**Table 2:** The energies (kcal/mol) of SO regioselective insertion in the ROAC of SO/PA catalyzed by different ureas.

	U1	U2	U3	U4
**INT2**	−11.6	−18.2	−17.0	−22.0
**TS3**	15.0	9.7	11.3	7.4
Δ*G*^‡^_(TS)_	26.6	27.9	28.3	29.4
**INT2′**	−11.5	−18.1	−17.4	−22.4
**TS3′**	15.7	10.5	12.1	8.6
Δ*G*^‡^_(TS’)_	27.2	28.6	29.5	31.0
ΔΔG^‡^	0.6	0.7	1.2	1.6

## Data Availability

Data are contained within the article and Supplementary Materials.

## Supplementary Materials

The following supporting information can be downloaded at: https://www.mdpi.com/article/10.3390/polym16070978/s1. All optimized coordinates are provided in the coordinates.xyz file.
